# Interactions between the mammalian main and accessory olfactory systems

**DOI:** 10.3389/fnana.2014.00045

**Published:** 2014-06-13

**Authors:** Michael Baum, Jorge A. Larriva-Sahd

**Affiliations:** ^1^Department of Biology, Boston University, Boston, MA, USA; ^2^Departmento de Neurobiología del Desarrollo y Neurofisiología, Instituto de Neurobiología, Universidad Nacional Autónoma de MéxicoQuerétaro, Mexico

**Keywords:** accessory olfactory system, main olfactory system, ordor, pheromones, sensory interactions

The controversial issue on the independent versus synergic functional role of the vomeronasal and main olfactory systems, motivated the present Research Topic (Larriva-Sahd and Baum, 2014). The resulting 11 studies fall into five broad categories: onto- and phylogenetic, structural, connectional, and integrative or review articles.

Two developmental works deal with the differentiation and distribution of neurons and glia in the main- (MOB), accessory-olfactory (AOB) bulbs, and primary olfactory cortex. The work by Pedraza and De Carlos (2012) presents the pallial and subpallial contributions to the primary olfactory cortex during the prenatal development, whereas Martín-López et al. (2012) describe the ontogenetic development of the MOB and AOB from reeler and wilde mice, with special reference to their laminar organization. In the latter study, cellular distribution over the course of prenatal development is further illustrated by both neuronal and glial markers. The cytological study by Larriva-Sahd (2012) concerns two, poorly-understood bulbar structures: the site of intersection between the MOB and AOB, or olfactory limbus (OL), and the alpha component of the anterior olfactory nucleus (aAON) that underlies the AOB. This work combines silver, and aniline stains, immunocytochemistry, and electron microscopy to define broad cellular organization, cellular types, and the neuropil of the OL and aAON.

The initial dogma regarding VNS and MOS as independent sensory modalities was supported by the dissimilar centripetal distribution of their tributary fibers. However, with the refinement of the tract-tracing techniques numerous sites of central convergence between the two systems have emerged. Three original studies performed in adult mice deal with the connectivity of the MOS and VNS. The paper by Hintiryan et al. (2012) provides a splendid atlas composed of colorful series of sections from brains that had previously been injected with antero-retrograde tracers. These unique specimens permit identification of the axonally linked areas that confirm previous connectional studies. Furthermore, the high resolution afforded by tiny sites of the tracer injections reveals novel interactions between the MOB and AOB with an unexpected detail. The connectional work by Mohedano-Moriano et al. (2012) is complementary in several ways, as it defines the second and third order targets of the MOB and AOB. Since non-sensory recipient areas, especially the nucleus of the diagonal band of Broca, project to both AOB and MOB, the possibility of a simultaneous supra-sensorial modulation of both systems is suggested. In this context (Pardo-Bellver et al., 2012) define the projections of the medial amygdaloid (MA) subdivisions, a nucleus that has been implicated globally in both the behavioral and endocrinological aspects of mammalian reproduction. The highly organized projections to the anterior, posterodorsal, and posteroventral subdivisions of the MA to amygdaloid, bulbar, septal, hypothalamic areas and nuclei, expand the notion that the MA itself is both structurally and functionally compartmentalized. The study by Gascuel et al. (2012) proposes the existence of a system of cells and fibers positive Orexin A-immunoreactivity (O-I) within the hypothalamus, basal forebrain and olfactory bulb itself. Since O-I fibers distribute within and between the hypothalamus and olfactory bulb, therefore author's results support the existence of an orexinergic system possibly associated with the olfactory influences on the diencephalic control of food-intake.

It is now clear that plasticity in the adult nervous system is more common than originally suspected (see Merkle et al., 2014). Notably, the adult MOB and AOB incorporate adult-born neurons to their preexisting circuitry. Rather than a steady generation and incorporation of prospective neurons to the bulbar cortex, this process is highly influenced by the concurring local and environmental demands. The investigation of Portillo et al. (2012) provides cytological and behavioral evidences supporting the notion that sexual experience in rodents enhances incorporation of adult-born cells to the accessory olfactory bulb.

Four review articles present and analyze current concepts of the MOS-AOS interaction. In this framework, the assays by Suárez et al. (2012) and Mucignat-Caretta et al. (2012) present parallel interactions between both systems as a function of species and phylogenetic evolution, respectively. Relevant from the comparative functional perspective is the work by Keller and Lévy (2012) providing direct evidence for the involvement of the main olfactory system in decoding environmental chemosignals from conspecifics. This decoding of socially relevant clues has traditionally been attributed to the AOS, but these authors offer a comprehensive analysis of species differences in this regard. Lastly, the detailed review by one of the Associated Editors of this series (Baum, 2012) emphasizes the structural, connectional and behavioral information relevant for functional interactions between the main and accessory olfactory systems.

## Conflict of interest statement

The authors declare that the research was conducted in the absence of any commercial or financial relationships that could be construed as a potential conflict of interest.
